# Accuracy of Non-Contrast MRI for the Detection of Hepatocellular Carcinoma: A systematic review and meta-analysis

**DOI:** 10.12669/pjms.38.3.5142

**Published:** 2022

**Authors:** Liping Lu, Xuming Pan

**Affiliations:** 1 Liping Lu, Huzhou Third Municipal Hospital, the Affiliated Hospital of Huzhou University, Huzhou, Zhejiang Province, China; 2 Xuming Pan, Huzhou Traditional Chinese Medicine Hospital Affiliated to Zhejiang Chinese Medical University, Huzhou, Zhejiang Province, China

**Keywords:** Hepatocellular Carcinoma, Magnetic Resonance Imaging, Meta-analysis, Validation studies

## Abstract

Non-contrast MRI is used for identifying patients with hepatocellular carcinoma (HCC), especially among high-risk patients with cirrhosis or chronic viral hepatitis. The accuracy of non-contrast MRI has been investigated with varying results. We performed this meta-analysis to consolidate the evidence on the accuracy of non-contrast MRI for the detection of HCC. We conducted a systematic search in the databases of PubMed Central, SCOPUS, MEDLINE, EMBASE and Cochrane from inception till November 2020. We used the STATA software “Midas” package for meta-analysis. We included 15 studies with 3,756 patients. The pooled sensitivity and specificity of non-contrast MRI for HCC detection were 84% (95%CI, 78%-88%) and 94% (95%CI, 91%-97%). The positive likelihood ratio was 14.9 (95% CI, 9.0-24.7) and the negative one 0.17 (0.12-0.23). The overall quality of the studies was high. We found significant heterogeneity based on chi-square test results and I^2^ statistic > 75%. Deek’s test showed the absence of publication bias. We found that non-contrast MRI has high sensitivity and specificity as a tool for detecting HCC. Studies exploring its accuracy in different ethnic populations are required to strengthen the evidence.

## INTRODUCTION

Most patients with hepatocellular carcinoma (HCC) are diagnosed when presenting advanced cancer stages, making it difficult for curative treatments to work.^1^ Prevention or treatment of hepatitis infection, regular surveillance to diagnose HCC early, and prompt management are the most effective ways to reduce the mortality of HCC.^2^ Surveillance strategies target patients with high risk of presenting HCC such as those with liver cirrhosis or viral hepatitis, and they help to increase the number of HCC patients diagnosed with a potentially curable disease, thereby improving the HCC-related mortality.^3^

The European Association for Study of Liver (EASL), American Association for the Study of the Liver Diseases (AASLD), and Asian Pacific Association for Study of Liver have recommended a surveillance strategy for patients with HCC risk factors with biannual ultrasonography (USG).^4^–^7^ However, the accuracy of USG for detecting HCC is unsatisfactory. USG is only 63% sensitive for diagnosing early HCC, leading to delayed diagnoses in more than one-third of patients.^8^ Hence, alternate diagnostic tools for HCC need to be explored.

Possible alternatives for HCC diagnostics include contrast enhanced magnetic resonance imaging (MRI) and non-contrast MRI with or without diffuse weighted imaging (DWI) as a screening tool.^9^ However, use of intravenous (IV) contrast agents such as gadolinium for enhanced MRI are not widely acceptable for undertaking large-scale screening programmes due to controversies related to deposition of gadolinium in the body tissues and to its high costs.^10^ Hence, non-contrast MRI may be a more accepted alternative to USG for the detection of HCC. But, no systematic effort to pool all the evidence and provide a final answer on its accuracy to detect HCC has made. Our aim with this study was to conduct a detailed literature search and to synthesize the outcome data from studies reporting the accuracy of non-contrast MRI for the detection of HCC.

## METHODS

### Eligibility Criteria

We have included the studies assessing the accuracy of non-contrast MRI amongst the patients suspected to have HCC irrespective of study design. The reference standards for HCC detection included histopathology, biopsy, and contrast-enhanced MRI. We excluded unpublished studies and grey literature.

### Search Strategy

We conducted an explicit, comprehensive, and systematic search on PubMed Central (PMC), SCOPUS, MEDLINE, EMBASE and Cochrane databases. We used the PubMed search engine to search the PMC and MEDLINE databases. We used the following set of medical subject headings (MeSH) and free-text terms to search the databases from inception until November 2020: “Magnetic Resonance Imaging”, “Non-contrast MRI”, “Validation Studies”, “Hepatocellular Carcinoma”, “Tumours of the Liver”, “Diagnostic Accuracy Studies”, “Liver Tumours”, and “Liver Malignancy”. We did not set language restrictions, and we manually reviewed the references of the identified articles.

Two authors independently performed the initial screening by checking the title, abstract, and keywords of papers in the search results, and they downloaded the relevant full-text publications. Then, the same two authors independently read the downloaded full-texts to include the studies meeting our eligibility criteria in the review.

### Data Extraction

Primary investigator extracted the data using a pre-defined data extraction form. The data extraction included: publication year, author information, country/residence, region, setting, participants, design, total sample size, details of non-contrast MRI procedure and technique, reference standard, average age, sensitivity, and specificity. Another investigator ensured the quality of the data entry procedure by double checking the entries before performing the analysis.

### Quality assessment of diagnostic accuracy studies-2

(QUADAS-2) tool was used to assess the risk of bias under the domains: patient selection, conducting and interpreting the index and reference standard tests, and outcome assessment flow and timing^11^ and graded all the studies as having low, high, or unclear risk of bias.

### Statistical Analysis

We pool the sensitivity and specificity indices of non-contrast MRI for the detection of HCC based on a bivariate meta-analysis. We calculated positive and negative likelihood ratios (LRP and LRN) and diagnostic odds ratio (DOR) for the utility of non-contrast MRI. Our results are reported on forest plots (pooled specificity and sensitivity), LR scattergrams (LRP and LRN) and Fagan’s plots (pre- and post-test probability of detecting HCC). We calculated the chi-square and I^2^ statistic to assess heterogeneity between the studies.

Additional subgroup analysis was performed based on the intent of imaging (diagnostic/surveillance), condition of study participants and use of DWI. We performed meta-regression to find out the source of heterogeneity. The covariates adjusted during the meta-regression were study design, country, sample size, intent of source imaging, mean age, and quality related factors. We used Deek’s test to assess publication bias. Sensitivity analysis was performed to assess the robustness of the study results. We performed all analyses using the STATA software Midas command package.

## RESULTS

**Fig.1:**
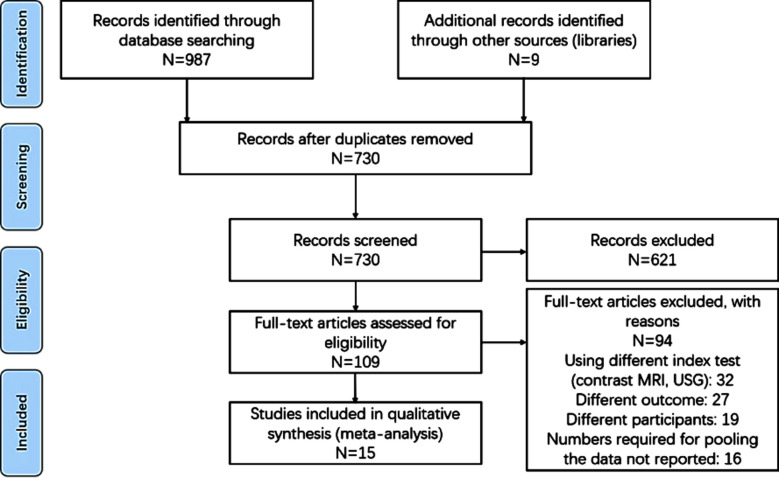
Search strategy.

We found 978 records through the systematic literature search, and deemed 109 of those studies relevant for full-text retrieval. We also retrieved full-texts for nine articles obtained through manual searching of the bibliographies. During the second screening stage, 15 studies with 3,756 participants met the eligibility criteria (Fig.1).^12^–^26^

Most studies (12 out of 15 studies) were retrospective. Almost half of the studies were conducted in Korea (7 out 15 studies). The average age of the patients ranged from 56 to 66.2 years. The MRI indications were almost equally distributed between diagnosis and surveillance, and most studies used histopathology/biopsy following surgery as the reference standard (Table-I).

**Table I:** Characteristics of the included studies (n=15)

Author, year, Country	Study design	Study participants	MRI Procedure and Intent	Reference standard	Mean age (in years)
Chan et al 2019^19^Australia	Retrospective	564 patients at high risk of HCC (cirrhosis, HBV/ hepatitis C virus/other risk factors) for HCC diagnosis	Non-contrast MRI study created by selecting axial T2- weighted sequence with 160-ms echo time, all four axial T1-weighted Dixon sequences	cMRI	63
Chung et al 2011^21^Korea	Retrospective	46 patients who underwent hepatic MRI for HCC surveillance	DWI on a single-shot spin-echo Echo Planar Imaging sequence that combined the two diffusion gradients before & after 180° pulse	Histopathology	59.6
Han et al 2018^25^Korea	Retrospective	247 patients with initial diagnosed HCC & no previous treatment history, within Milan criteria for HCC diagnosis	Liver MRIs on a 3T system with an 8-channel phased array torso coil. Respiratory triggered fast spin echo T2WI with fat suppression & dual gradient echo T1WI using in-phase and opposed-phase	Histopathology/ cMRI/FU	59.6
Hardie et al 2011^15^USA	Retrospective	37 patients who had undergone liver transplantation for HCC diagnosis	DWI on a single-shot echo-planar imaging with repetition time/echo time 4800/94; matrix 192¥100; parallel imaging factor 2; gradients with b-value 50, 500, 1000 s/mm^2^	Histopathology	56.6
Jalli et al 2015^13^Iran	Prospective	96 cirrhosis patients referred to gastroenterology follow-up for HCC diagnosis	Respiratory triggered single-shot fat-suppressed echo-planar DWI sequence in axial plane with acquisition correction on TR/TE, 2100/85 ms; 6mm slice thickness; with b value 50, 400, 800 s/mm^2^	Histopathology	NA
Kim et al 2014^22^Korea	Retrospective	182 patients with chronic hepatitis or liver cirrhosis for HCC diagnosis	DW-MRI single-shot echo planar imaging and simultaneous respiratory triggering on TR/TE 1600/70. b-value of 0, 100, 800 s/mm^2^; SENSE acceleration factor, 4.0; field-of-view, 35×35 cm;	Histopathology	57
Kim et al 2020^16^Korea	Retrospective	226 patients with a history of cirrhosis or chronic liver disease who underwent MRI of the liver for HCC surveillance	Liver MRI scans on a 3T system with a16-channel phased-array torso coil. Respiratory-triggered fast-spin echo T2WI with fat suppression and dual-gradient echo T1WI. DWI with echo planar imaging using b values of 0, 50, 400, 800 s/mm^2^	Histopathology	60.1
Min et al 2018^14^Korea	Retrospective	483 patients who underwent surveillance after hepatectomy	MR images on a 3.0 T whole-body MR system with a 16-channel phased-array coil as the receiver coil.	Histopathology	58
Park et al 2012^20^USA	Retrospective	52 patients who underwent liver transplantation for HCC surveillance	Liver MRI on different state-of-the-art 1.5-T systems and torso phased-array coils. Parallel imaging and field of view of 300-400 mm	Histopathology	56
Park et al 2020^23^Korea	Retrospective	1057 patients > 20 years diagnosed histologically/ radiologically as having cirrhosis with HCC surveillance	MRI on a 1.5-T scanner. Breath-hold dual gradient-echo T1-weighted images, DWI with a respiratory triggered turbo spin echo, single-shot echo planar sequence images with b-values of 0, 50, 500 s/mm^2^	Histopathology and radiologic hallmark	56.4
Shankar et al 2016^24^India	Prospective	20 patients presenting to hepatology clinic with chronic liver disease for HCC diagnosis	Abdominal MR on 3T imaging system using a body coil. The protocol included T1, T2 weighted axial imaging including both non-fat and fat-suppressed sequence, axial DWI	Cytological grading	NA
Sutherland et al 2016^17^Australia	Prospective	192 patients > 18 years referred by gastroenterology with chronic liver disease for HCC surveillance	MRI scan sequence comprised respiratory-gated DWI with TR 2500; TE 80; slice thickness 8 mm; distance factor 30%, FOV read 400 mm, and b values of 100, 400, 800	Histopathology	58
Violi et al 2020^18^USA	Retrospective	237 patients (≥ 18 years) with cirrhosis, chronic hepatitis B for HCC surveillance	Non-contrast MRI, including axial non-fat-suppressed T2WI single-shot echo-planar imaging + axial fat suppressed DWI single-shot echo-planar imaging	Histopathology	58
Whang et al 2020^12^Korea	Retrospective	263 patients with liver cirrhosis or other risk factors without prior history of HCC treatment	All MR images were acquired using 3.0-T MR system. Respiratory-triggered fast spin echo T2WI with fat suppression, 3D dual gradient echo T1WI using in- and opposed-phase. DWI with echo planar imaging using b values of 0, 50, 500, 800 s/mm^2^	Histopathology	64
Xu et al 2010^26^ China	Retrospective	54 patients who had undergone routine c-MRI & DWI before surgery for HCC diagnosis	All MR examinations were performed on a 1.5-T superconducting scanner with combination of a 6-channel phased-array body coil & spine array coil	Histopathology	66.2

We found that nine out of 15 studies had a high patient selection bias risk, six had a high conduct and interpretation of index test bias risk, 5 had a high patient flow and interval between index tests and reference standards bias risk, and none had a high reference standard bias risk.

**Fig.2:**
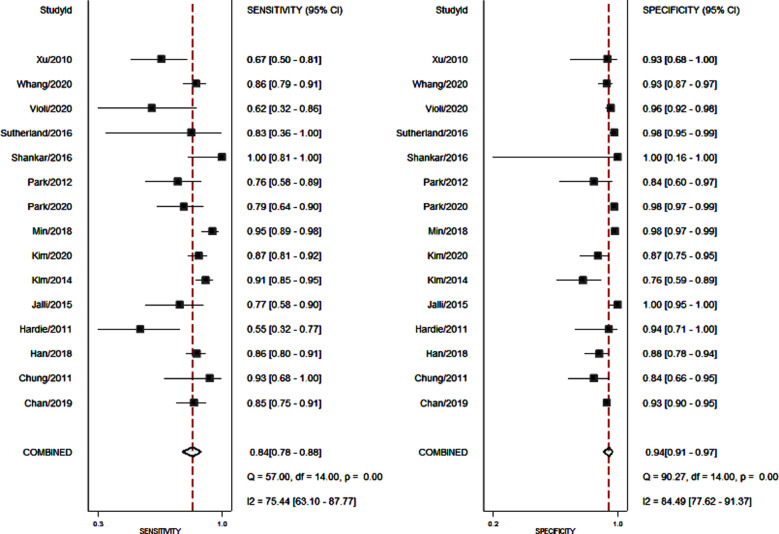
Forest plot showing pooled sensitivity and specificity for non-contrast MRI.

**Fig.3:**
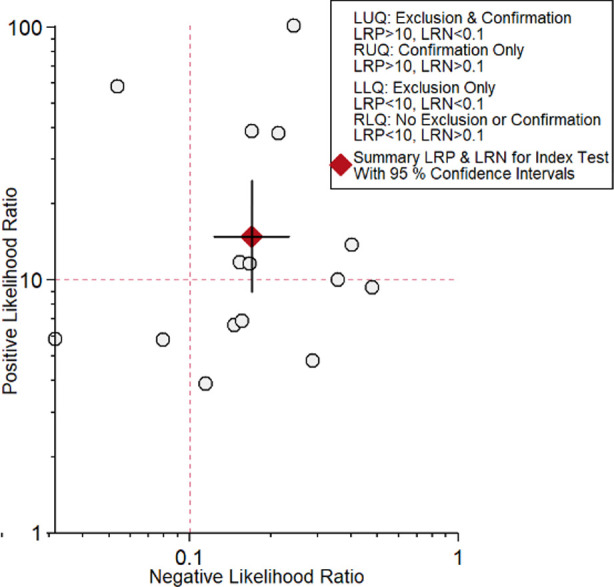
Likelihood scatter gram.

**Fig.4:**
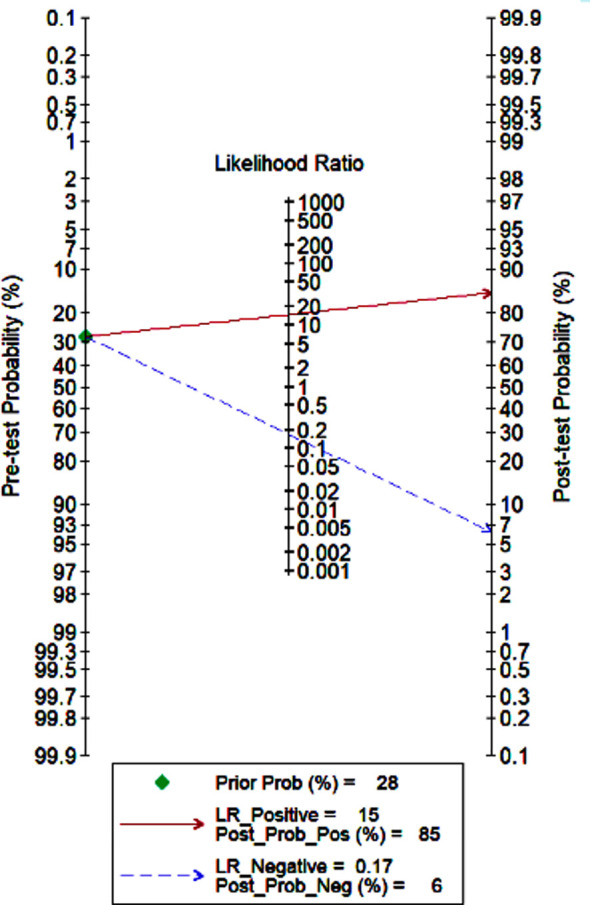
Fagan nomogram

The utility of non-contrast MRI for the detection of HCC was reported in 15 studies.^12^–^26^ The pooled sensitivity and specificity of non-contrast MRI for HCC among high-risk patients were 84% (95% CI, 78%-88%) and 94% (95% CI, 91%-97%), respectively (Fig. 2). The DOR was 87 (95% CI, 47-160). LRP was 14.9 (95% CI, 9.0-24.7) and LRN was 0.17 (0.12-0.23). The LR scattergram (Fig.3) shows that the LRP and LRN are in the right upper quadrant indicating that non-contrast MRI can be used for confirmation only. Fagan’s nomogram (Fig.4) shows a high clinical utility of non-contrast MRI for HCC detection (Positive=85%; Negative=6%) differing significantly from the pre-test probability (28%). We also found significant heterogeneity with chi-square p-value<0.001 and I^2^=91%.

Subgroup analysis based on intent of imaging showed that the non-contrast MRI is more accurate when it is used as surveillance tool (pooled sensitivity=85% & specificity=95%) than diagnostic tool (pooled sensitivity=83% & specificity=93%). Sensitivity and specificity among the cirrhosis/chronic hepatitis/chronic liver condition patients were 84% and 95%. Use of DWI imaging did not significantly differ the sensitivity (82%) and specificity (95%) compared to overall estimates.

Meta-regression results indicate that index test standards (p<0.001) and flow and timing of tests (p<0.001) was the sources of heterogeneity in sensitivity model, study design (p=0.04) and patient selection (p=0.03) were significant in specificity model, and mean age was the source of heterogeneity in the joint model (p<0.001). Deek’s test showed a non-significant p-value (p=0.21) indicating the absence of publication bias. Sensitivity analysis revealed there was no significant single study effects or effect from inflated pre-test probability on the accuracy of the non-contrast MRI for HCC.

## DISCUSSION

The MR imaging system is used for identifying patients with HCC, especially among those with high-risk of developing the malignancy (patients with cirrhosis and chronic hepatitis infection). Non-contrast MRIs can be used as they are less time consuming, easier to obtain, carry lower healthcare costs, and prevent the harmful effects associated with the contrast agents on dynamic contrast enhanced MRIs. However, the evidence of their utility has not been synthesized. Hence, our goal with this review was to determine the accuracy of non-contrast MRI for the detection of HCC, especially amongst high-risk patients.

After the systematic literature search, we found 15 studies (most retrospective in nature and with low bias risks) reporting the utility of non-contrast MRI for HCC. We found a high pooled sensitivity (84%) and specificity (94%) for non-contrast MRI in HCC detection. Other accuracy parameters also showed a high accuracy: in the LR scattergram, LRN and LRP occupied the right upper quadrant indicating that the imaging technique can be used for both HCC confirmation. The clinical utility of non-contrast MRI was also significantly better as the Fagan’s nomogram showed a significant rise in the post-MRI probability compared to the pre-MRI probability.

The accuracy parameters for the contrast-enhanced MRI we obtained in this review are similar to those reported by Roberts et al (2018) for the same technique and better than those for contrast-enhanced CT scans in that same review.^27^ Another review assessing diffusion weighted MRI also showed accuracy parameters similar to ours.^28^ We also found that non-contrast MRI acts as a better surveillance tool than being a diagnostic tool for HCC. We also tried to assess the impact of chronic liver conditions, and use of DWI on the accuracy of the non-contrast MRI. We found that there was no change in the specificity and mild reduction in the sensitivity in case the patients present with cirrhosis/chronic hepatitis/any chronic liver conditions. Similar finding was observed for the use of DWI in the imaging system. Further updated reviews should compare the performance of non-contrast MRI with other similar imaging techniques.^29^

However, our results need to be interpreted and inferred with caution, considering the quality and difference in methods among the included studies. For example, we found significance between-study variability. This heterogeneity can be attributed to the varying ethnicity of the study participants and to the differing risk factors and severity amongst the patients in the studies included. Deek’s test results and the funnel plot pointed to the absence of publication bias. In addition, studies like Chung et al. 2011^21^ included only hypervascular lesions and Hans et al 2018^25^ including only the initial HCC diagnosed patients leading to marked inflation in the pretest probability of HCCs in this cohort compared to general pool of at-risk patients. However, sensitivity analysis performed by excluding these studies also revealed sensitivity (83%) and specificity (95%) to be high.

Our review is the first meta-analysis assessing the accuracy of non-contrast MRI for the detection of HCC among high-risk patients, and it involved a large number of studies with high sample sizes. Most included studies showed high QUADAS-2 tool quality, and we found no significant publication bias adding to credibility of our meta-analysis.

### Limitations of the study

First, we found a significance between-study variability in our analysis that limits our ability to infer or interpret the pooled findings. Second, the accuracy of the non-contrast MRI depends on multiple factors including some which we could not assess like the ethnicity, timing of the non-contrast MRI assessment, and severity and risk factors of the patients.

## CONCLUSION

Our findings suggest that non-contrast MRI can be used for the detection of HCC. The use of non-contrast MRI in these patients can help in reducing the time spent with diagnostic procedures and also the healthcare costs. Large-scale setting-specific longitudinal studies are required to establish non-contrast MRI as the standard assessment tool for all the high-risk patients.
